# Exploring the experience of cardiothoracic ICU clinicians during the COVID‐19 pandemic: A grounded theory study

**DOI:** 10.1111/nicc.13186

**Published:** 2024-10-25

**Authors:** Leah Hughes, Benjamin Shelley, Joanne McPeake

**Affiliations:** ^1^ NHS Ayrshire and Arran Ayr UK; ^2^ Golden Jubilee National Hospital Glasgow UK; ^3^ School of Medicine, Dentistry and Nursing University of Glasgow Glasgow UK; ^4^ The Healthcare Improvement Studies Institute University of Cambridge Cambridge UK

**Keywords:** cardiothoracic, COVID‐19, health care staff, moral injury, visiting

## Abstract

**Background:**

Prior to the COVID‐19 pandemic a flexible approach to visiting was adopted by many Intensive Care Units in the United Kingdom. Due to the rapid spread globally of COVID‐19, significant policy changes were put in place, including the restriction on visitors to patients in hospital. Evidence has emerged demonstrating the negative impact of these restrictions on patients with COVID‐19, their families and the staff caring for them. However, there is limited data about the impact of these restrictions in the non‐COVID ICU environment.

**Aim:**

This study aimed to explore the experiences of staff caring for non‐COVID‐19 patients in a cardiothoracic critical care unit during the COVID‐19 pandemic.

**Study Design:**

This qualitative research study adopted a grounded theory methodological approach. This methodology was used due to the unique situation, with no prior research available. We recruited healthcare staff that worked in a cardiothoracic critical care unit during the COVID‐19 pandemic. Semi structured interviews were carried out, transcribed, and analysed. Using the data collected, a theory was constructed.

**Results:**

Interviews were carried out with 20 healthcare staff from a range of professions including nurses, doctors, and allied health professionals. Following data analysis four main categories emerged from the data: impact and implementation of visiting restrictions; the dehumanisation of patients; end‐of‐life care and witnessing distress. From these four categories, a theory has emerged suggesting that healthcare staff in a non‐covid ICU were regularly exposed to potentially moral injurious events, despite being shielded from caring for patients with COVID‐19.

**Conclusions:**

This study provides a theory that healthcare staff caring for non‐COVID‐19 critical care patients during the period of visiting restrictions were exposed to potentially morally injurious events.

**Relevance to clinical practice:**

Repeated exposure to potentially morally injurious events can lead to the development of moral injury and its adverse consequences. This study highlights the need to support all staff in the post COVID era, including those who worked in a non‐COVID environment.

**What is known about the topic**

- During the height of the COVID‐19 pandemic, hospital visitation was severely restricted in order to reduce transmission.
- There are limited data published on the impact of COVID‐19 visiting policies in the non‐COVID ICU setting.

**What this paper adds**

- The findings from this qualitative study suggest that those working in the ‘non’‐COVID‐19 setting during pandemic were regularly exposed to Potentially Moral Injurious Experiences, despite being relatively shielded from caring for those with COVID‐19.
- Priority should be placed on supporting all staff in the post‐pandemic era; this should include prioritizing research into the management of moral injury.

## 1 INTRODUCTION

In early 2020, reports of a novel coronavirus (COVID‐19) were emerging from China. Within weeks, cases of COVID‐19 were being identified globally and the World Health Organization (WHO) declared a pandemic.^1^ The rapid spread of COVID‐19 led to significant policy changes in health care, including changes to hospital visitation policies.^2^, ^3^ In the UK, for example, hospital visiting was limited to special circumstances such as end‐of‐life care, supporting a birthing partner and supporting those with additional needs in acute care.^4^

## 2 BACKGROUND

Prior to the COVID‐19 pandemic, the importance of visitors in the ICU was recognized,^5^ with around 80% of ICUs in the UK offering more than 4 h of visiting per day.^6^ It is understood that visitors are a valuable resource, not only providing psychological support to patients but there is also evidence to suggest that the presence of visitors is associated with other benefits, such as the reduction in the occurrence of delirium in the ICU.^7^, ^8^ As such, the near complete absence of family members and visitors was a stark change to the ICU environment.

Since the implementation of COVID‐19 visitation policies, research has emerged demonstrating the negative impact these policies have for patients, their families and staff caring for them.^2^, ^9^ For example, in one French multicentre study investigating the experience of clinical staff caring for patients with COVID‐19, participants described adverse symptoms of acute stress, including that of insomnia and developing nightmares.^10^ To date, however, the focus of the evidence base has been on patients admitted with COVID‐19 and those staff caring for them. The impact on the wider ICU system has not been sufficiently explored, with limited data published on the impact of COVID‐19 visiting policies in the non‐COVID ICU setting.

Therefore, using a qualitative approach, we sought to explore the experiences of ICU clinical staff and the impact of the visiting restrictions in the non‐COVID ICU setting.

## 3 AIMS AND OBJECTIVES

This study aimed to explore the experiences of staff caring for non‐COVID‐19 patients in a cardiothoracic critical care unit during the COVID‐19 pandemic and develop a preliminary theory in relation to how these experiences manifested.

## 4 RESEARCH QUESTION

What were the experiences of health care staff and the impact of visiting restrictions in the non‐COVID ICU?

## 5 DESIGN AND METHODS

### 5.1 Setting and sample

This study was conducted in a single tertiary referral centre in Scotland. During the research data collection period, this centre predominantly treated patients undergoing elective and non‐elective cardiothoracic surgical procedures, cardiac transplantion, acute heart failure treatment and out‐of‐hospital cardiac arrest care. In addition, some non‐cardiothoracic surgical patients were also admitted to the unit in this period. This centre was maintained as a ‘COVID light’ centre throughout the study period; as such, most patients admitted did not have COVID‐19 as a primary diagnosis.

All health care staff, including all allied health professionals and clinical support workers, over the age of 18, working in the critical care department during the period of visiting restrictions were invited to take part. Initially, invites were sent out via email by the researchers. Written information was sent out to those who expressed an interest in taking part, and they were given time to read the information and decline participation if they wished. Following this, an initial purposive sampling strategy^11^ was adopted in order to address the aims of this research. This allowed the researchers to recruit participants from various roles and grades within the multidisciplinary team and invite participants from staff groups which were under‐represented, ensuring that the study population had diversity of gender, age and profession within it. Although grounded theory adopts theoretical sampling as an approach to recruitment,^12^ it is widely recognized that purposive sampling is suitable and is a realistic approach at the initial stages of data collection.^13^

### 5.2 Data collection and methods

This study was conducted using semi‐structured interviews guided by Charmaz's methodology of constructivist grounded theory.^10^ Grounded theory was adopted as it was felt to be the most appropriate method to uncover relationships and behaviours, and would allow the researchers to transform exploration into a theory.^11^ Data were collected systematically and analysed concurrently, during which the theory was developed.^14^, ^15^ The data collected were used to construct a theory about the effects of visiting restrictions on health care staff.

Semi‐structured interviews were carried out by one researcher (LH), an experienced Advanced Critical Care Practitioner.^16^ Interviews were digitally recorded and transcribed verbatim while field notes were created by the researcher following each interview. Open‐ended questions were used to enable participants to describe and elaborate on their experiences of caring for patients during the COVID‐19 pandemic. The initial interview guide, found in Appendix 1, was adapted as data collection progressed. In order to provide data verification, two participants reviewed the transcript of their interviews, confirming they were accurate. Ethical approval was granted to interview 20 participants. This sample was chosen to allow diversity among the participants and ensure a theoretical approach to recruitment could be achieved. As such, not all volunteers were recruited to the final study population, and this allowed for the data collected to direct which participants should be recruited, allowing a more theoretical approach to sampling to occur; it also had the benefit of ensuring that all professions working clinically during the research period were represented in the study population. Initially, participants from a wide a range of roles and experience were recruited. As data underwent initial coding, the data itself guided further recruitment of participants. This process continued alongside the concurrent analysis of data.^11^ The researchers (JM and LH) agreed when data saturation had been achieved. Data saturation occurred when no new significant categories emerged from the data. After 20 interviews, it was felt that saturation had occurred, and there would be no advantage from further study recruitment.

### 5.3 Data analysis

In line with Charmaz's approach to grounded theory,^12^ coding was carried out in stages. First, initial coding was carried out line by line, and then in larger sections or sentences. This identified subcategories in the data. The second stage of data analysis used focused coding. Memo development and constant comparison of the data occurred simultaneously alongside the coding process. Categories were developed, leading to the construction of the primary grounded theory. Once no new codes were identified from the data, it was felt that data saturation had occurred. In order to maintain an audit trail, field notes were taken, memos were constructed and maintained within a database alongside anonymised supporting quotes.

The first author (LH) carried out data analysis, with frequent discussions with JMcP, an experienced qualitative researcher aware of the processes involved in grounded theory research, who was familiar with the data. Both LH and JMcP took part in the process of developing the theory.

## 6 ETHICAL AND RESEARCH APPROVALS

Ethical approval was obtained from the Yorkshire & The Humber—South Yorkshire Research Ethics Committee (20/YH/0236) prior to recruitment. All participants received both written and verbal information about the study prior to giving written consent. All interviews were anonymised after transcription.

## 7 FINDINGS

We conducted 20 semi‐structured interviews with health care staff between October and December of 2020. Health care staff from a range of professions and roles took part in this study (Table 1). Five men and 15 women participated. The median age of participants was 40 years (IQR: 32–50) and the median length of professional experience was 16 years (IQR: 8–26).

**TABLE 1:** Participant demographics.

	*N* = 20	
Age		
Median (IQR)	40	(31.5–49.5)
Range	27–59	
Gender		
Male, *n* (%)	5	(25)
Female, *n* (%)	15	(75)
Years of experience		
<5, *n* (%)	2	(10)
6–10, *n* (%)	5	(25)
>10, *n* (%)	13	(65)
Median (IQR)	13	(7.75–25.75)
Profession		
Medical, *n* (%)	5	(25)
Nursing, *n* (%)	9	(45)
Allied health professional,^a^ *n* (%)	6	(30)

^a^ Allied health professionals include physiotherapists, speech and language therapists, dieticians, occupational therapists and support staff.

Four categories were developed from the data collected: impact and implementation of visiting restrictions; the dehumanization of patients; end‐of‐life (EoL) care; and witnessing distress. The following sections describe these categories in detail alongside illustrative quotes. Further illustrative quotes can be found in Table 2.

**TABLE 2:** Illustrative quotes.

Category	Supporting Quotes
Impact and implementation of visiting restrictions	‘I think we are isolated as well in that we are in the room*…* But the isolation definitely impacts us because we're not getting that interaction with our colleagues*…* I think it impacts on your mood and on your morale*…*’ (S13)
	‘But I personally feel as if I'm kind breaking the rules and while I feel able to make that decision and I'm doing it because I think it's genuinely the right thing to do there's always that worry of will you get into trouble for having a visitor *…* quite often that decision will fall to myself *…*’ (S19)
The dehumanization of patients	‘I think the relatives humanize the patients, they humanize us, they remind us that this patient has an existence out with this hospital. They remind the patient they have an existence out with this hospital.’ (S05)
	‘It just hasn't been the nursing philosophy that you have throughout your career *…* to go from supporting relatives as well as patients to then feeling like you're a barrier to that, you're separating relatives from the people they love actually in the early months weighed heavy in terms of guilt for me*…*’ (S20)
End‐of‐life care	‘*…*usually the patient's condition is deteriorating so you would have met with the relatives in the lead up, in the days leading up to that and would be saying to them “this isn't going so well, this is a likely outcome.” And so you do some preparation and some groundwork I guess, and usually it makes the end of life conversation a little bit easier, I think it just comes out of the blue basically which is always tumultuous and upsetting for the relatives.’ (S07)
	‘So how on earth can I ask a family to decide who the person that doesn't get to see him? And I take on a future concern of how are those two sisters are going to make their peace with knowing that I got to see dad and you didn't? And the fact that we put them in that position in the first place.’ (S20)
Witnessing distress	‘…but his son had never seen him and was totally devastated and horrified because he's so gaunt and he's lost so much weight*…* even though he's progressed and we think he's doing great, it's still a horrendous situation to see someone that you care about in. I don't think you can ever prepare for that so.’ (S08)
	‘But by far one of the most upsetting things I've seen during this is a relative come to the door with somebody they loved and not being able to step further than the door*…* I find that heart‐breaking *…* The idea of just dropping someone off and leaving them, it was terrible.’ (S20)

### 7.1 Impact and implementation of visiting restrictions

Participants described the emotional burden of the implementation of visitation guidelines in practice. As guidelines were developed nationally, those implementing the guidance had no influence or insight into policy development. Some participants displayed frustration at the application of this policy, expressing that frontline clinicians should have been allowed a degree of autonomy regarding decision‐making around visitation.

I think the clinicians working in the Unit should have more autonomy to make decisions related to the patients and their families, and by that I mean I think the clinicians working in the Unit should be the ones to manage who comes up and when, because I think we're the ones that are looking after these patients. We're the ones that are looking after these families. We're the ones that have to tell them, ‘sorry you can't come up and see your dad who's dying’, and I think the burden is on us. [S11]

This depiction of the burden placed on health care staff highlights the negative effects visitation policies had on the clinical staff implementing them. The application of policy caused distress. Frontline staff were required to manage and implement policies which often resulted in family anger and distress. This led to challenging situations, and participants displayed guilt about their involvement in implementing the guidelines.

It has been quite difficult but we're on the front line here and we're the ones that are saying, it's not you know the politicians who are saying no, it's us who are saying no to these people and we're the ones taking the brunt of somebody's anger. [S17]

This lack of control with regard to decision‐making, and the repeated exposure to the anger and distress of relatives when implementing the rules, had the potential to expose health care staff to recurrent distress. Some participants described that senior staff used judgement in the implementation of the guidelines, taking on the burden of the decision‐making. This often led to an inconsistent approach in their implementation, resulting in other health care staff having to deal with the repercussions of the previous decisions made by others. This caused further stress.

I say, “Well yes okay I'll let somebody in and this is what's to happen,” or “Well there was two allowed in last week, and you only let one in, why you only let one in?” Because that's the latest rules and two probably shouldn't have been in but then I wasn't there so I can't see why that decision was made at that time. It's hard because you're trying not to‐ You don't want relatives to think that it depends who's on. [S19]

Participants interviewed also described that there was a perceived increased workload because of the absence of visitors. Participants (*n* = 9) discussed the increased time spent speaking with families on the telephone; it was recognized that this impacted nursing staff most. Participants from the Allied Health Professions also discussed adjustments to their role; most notably, they found therapies took longer to deliver, because of the need to provide additional support to patients.

I mean there's times where I might just spend five minutes, ten minutes just talking to the patient about the football on the weekend just because they want to talk about it and they've got no‐one else to talk about it with and I will, if I've got a spare five minutes, I'll just have that normal social conversation with them rather than it being purely clinical… [S06]

Participants described the impact of visitation restrictions on isolation. Isolation was not just experienced by health care staff; participants discussed their experiences witnessing the effects of isolation on both patients and their relatives. Physical barriers increased both health care staff and patients' exposure to isolation: these included the impact of personal protective equipment (PPE) as a barrier.

A lot of people feel even if you have that on that you can't you know hold somebody's hand… it is very difficult and some people are feeling it is a big barrier to them to actually physically sit down and hold somebody's hand and you know touch their arm and things like that. [S17]

This highlights the difficulty in providing comfort to patients not solely based on the requirement for PPE, but also a mental barrier to providing comfort to patients. The recurrent nature and lack of control experienced by participants when implementing guidelines increased the levels of distress experienced.

### 7.2 The dehumanization of patients

Participants described the change in the relationship between staff and patients because of the lack of family member presence. Families play a pivotal role in providing background to the patient as a person before their critical illness. Participants highlighted that they understood the patient's clinical needs intimately, but non‐clinical information about the patients was lost, with an ultimate loss of human connection.

So it's just kind of, it's not being able to put more, you're almost treating the body rather than the mind as well because sometimes if they're intubated or they're just starting to become alert or they're delirious, you don't really know what their, their sort of thought processes are, what their values are … [S09]

This loss of human connection had further implications for the participants. It not only affected the relationship between staff and patients but also how staff felt about their role and the satisfaction it gave them.

I guess it's taken away some of the joy… knowing the person, yes, knowing the family, and the extended family and having the grandkids in the room and all of that. [S14]

I think the relatives humanise the patients, they humanise us, they remind us that this patient has an existence out with this hospital. They remind the patient they have an existence out with this hospital. (S05)

Health care staff's requirement to understand the patient as a person correlates with their fulfilment in carrying out their clinical role. Without visiting, for many patients in ICU, this connection is lost and potentially impacts staff wellbeing.

Importantly, it was not just the relationship between patients and health care staff that was impacted by visitation restrictions. The restrictions also had a negative impact on the relationship between health care staff and relatives.

I hate not being able to see their faces and I hate them not being able to see mine and I think it's very sad that we could be together on a phone call for days or weeks and then we could walk past each other in the supermarket. We wouldn't know who each other was. [S20]

This highlights the importance of speaking face to face with relatives and also plays a part in the staff's perceived value in the care of patients. There was a loss of professional identity; relatives appeared to have a diminished understanding of professional roles within the multidisciplinary team. This loss of professional identity had the potential to decrease job satisfaction.

…our physio role in the eyes of the family is probably reduced because they'll be speaking to the doctors, they'll be speaking to the nurses and that will be the, their kind of prime focus on how their family member is doing. Whereas they might not hear the focus of how much rehab they're getting, and kind of how our profession stands in terms of significance within the, our role within the M. D. T. [S06]

This dehumanization and loss of connection with patients and their relatives has the potential to negatively impact health care staff, reducing job satisfaction and in turn increasing their exposure to distress within the workplace.

### 7.3 End‐of‐life care

Exposure to the distress of others while delivering EoL care was identified and developed as a highly emotive category. For some participants, the implementation and the change in how EoL care was delivered to patients and their families had a significant impact on them.

The way that we have worked for years in trying to support families and to deliver good end of life care, not just to patients but to the families as well, to support them through difficult times. This is just completely, it's almost barbaric for us to process this… [S11]

Across the interviews, there were clear signs of distress when participants discussed EoL care. It was acknowledged that although restrictions were in place, a concerted effort was made to allow families to be present if death was felt to be imminent. Participants recognized that EoL is not a clearly defined period for patients, and this led to staff allowing visiting, but with uncertainty as to whether these visits complied with the approved visiting exemptions. This uncertainty in decision‐making caused anxiety for staff.

I think because there was sometimes uncertainty what, we didn't know what was quite going to happen and we thought it was important that they got a chance to see their partner…to see their loved one, because we didn't know if they were going to recover or not and I don't know if that was strictly within the rules. [S08]

Restrictions required limited numbers of visitors at the EoL, and participants discussed the need for families to choose which family member could visit. Participants described the emotional distress involved in these conversations and situations with families:

… and then when it, it was inevitable that he was going to pass away, they were then left with the awful decision of who was going to come to the hospital and one of his sons will never have had the chance to say goodbye. [S11]

This exposure to the substantial distress by families and lack of ability to alleviate the distress to the family was a burden placed on the staff involved.

Conversations between health care staff and family were difficult during the EoL period. There was a change in how EoL conversations developed; families could not be prepared in the same manner as had been the norm before the visitation restrictions.

…usually the patient's condition is deteriorating so you would have met with the relatives in the lead up… And so you do some preparation and some groundwork I guess, and usually it makes the end of life conversation a little bit easier… (S07)

One participant described an EoL conversation as a professional low point and acknowledged the burden these conversations had.

it's probably my professional low point to phone a relative of somebody who was dying in intensive care and tell them I was very sorry they were dying and …they could come in, but if they came in, they wouldn't be able to go to the funeral. [S12]

Conflict between the wishes of relatives, representing potentially those of the patient, and the wishes of the health care team arose on occasion. Witnessing conflict and the distress of the family was upsetting for all involved and had a significant emotional impact on staff:

We should have been making decisions based on her family. They were telling us what she would like, and I almost thought the doctors saying, ‘but let's just try another day, but let's just try this.’ I think there's a bit of fear about making decisions because the families are not getting involved. So I found that quite difficult… not knowing this was going to end, knowing her family consistently saying, she would not want this. And we were still going on. So I think if the family had been here maybe they could have had more of an impact on the doctors' decisions. [S13]

### 7.4 Witnessing distress

The distress witnessed was not confined to the EoL period. One participant spoke of a conversation they witnessed between a patient and their family following major surgery.

…because it's hard to see, you know, and it's hard to hear, you know, patients on the phone saying that they…. They miss their relatives… [S03]

This demonstrated the concern that the participant felt when witnessing these potentially emotional conversations between patients and their relatives. Participants described attempting to improve the experience for patients and reduce their distress.

And I think as a staff member it definitely has an impact. There's a couple of patients who I definitely feel should have had family visits. And I feel ethically actually I think it's wrong. And that does have an impact on yourself because you feel kind of empathy for the patient. You feel sad for the patient that they've not got that support. But you also feel that you almost need to provide more support in your role to try and kind of overcome some of the detriment of the fact that they've not had family members in. [S15]

This need to provide extra support to patients increased the toll on staff. Some participants attempted to alleviate the distress of patient isolation, often taking on the role of a family member; however, other participants acknowledged that was a role that was not only challenging to undertake but potentially not achievable as health care staff should not take on the role of the family member.

I think that's particularly difficult for staff. And if you've got somebody who is really sick, it would be nice to know if they can hear what's going on, that their wife is just talking to them. And just holding their hand. And as much as we can reassure them and talk to them, we are not their family. [S13]

Participants were exposed not solely to the distress of the patients in their care but also to the distress of relatives. Participants spoke at length about the way in which they communicated with relatives had changed. While not all changes were negative, the increased exposure of relative distress and the negative emotional impact for staff was evident:

…we used to obviously give minimal information over the phone and you would be waiting for a family member to come, so that you could fully update them. You know you're now updating some really hard stuff over phones or over FaceTime and sometimes it's quite hard, you know, you always say things wrong, but you only have that verbal communication, you're not able use any other way of communication… [S04]

The fear of making mistakes when updating families and the implications of this was apparent in the data.

There was an acknowledgement that there was an increased burden placed on health care staff because of visiting restrictions. Some recognized that there were no strategies in place to provide psychological support for staff. Participants described the experiences of colleagues struggling with the added burden of visiting restrictions.

I think there's a lot of people who perhaps can't cope with having the psychological burdens so they maybe don't have a mechanism of sharing that and offloading that to other people so it's about not getting overburdened by relatives' or patients' psychological needs because it can be quite hard work sometimes and staff feel a bit overwhelmed… [S19]

This highlights that health care staff not only experienced emotional distress themselves but also witnessed it in their colleagues, with no ability to alter or improve the situation to alleviate their own or their colleagues' suffering.

### 7.5 Exposure to potential moral injury

**FIGURE 1:**
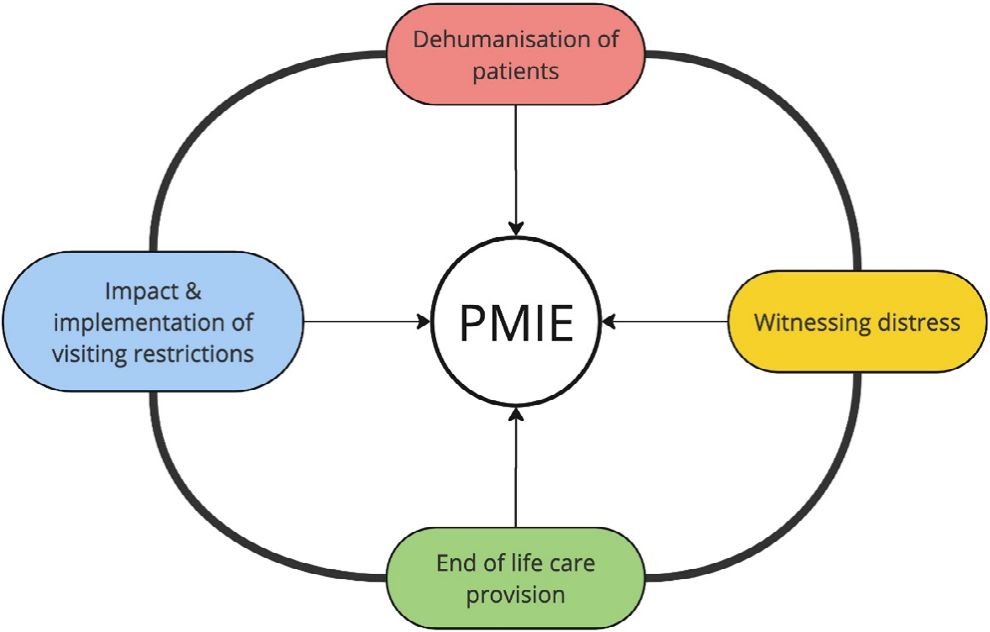
Theoretical framework: Factors contributing to potential moral injury.

The major categories to emerge from the data were the impact and implementation of visiting restrictions, the dehumanization of patients, EoL care provision and witnessing distress. These categories were not independent, and there was overlap between them. This can be seen for instance in the EoL care provision category and witnessing distress categories. Based on these findings and the development of these categories, the theory that health care staff experienced recurrent exposure to Potentially Moral Injurious Experiences (PMIEs) was formed (Figure 1). PMIEs are experiences that are either executed by, failed to be prevented by or witnessed by an individual that goes against their own moral beliefs.^17^, ^18^ Across the data collected, participants repeatedly described exposures to events outside the bounds of their own control, but with the burden of implementation placed on them. As a result, the theory that health care staff working in non‐COVID ICUs experienced recurrent exposure to PMIEs emerged.

## 8 DISCUSSION

This analysis explored the experiences of clinical staff working in a non‐COVID ICU during the pandemic, focusing on the impact of visiting restrictions as experienced by staff.

Regular exposure to PMIEs is believed to contribute to the development of moral injury.^17^ Moral injury is widely accepted in military medicine, and prior to COVID‐19, it was increasingly accepted as an occupational hazard to those working in health care.^18^, ^19^ It is associated with feelings of guilt, anger or disgust and is linked with distress psychological issues. It is also recognized that moral injury often co‐exists alongside post‐traumatic distress syndrome (PTSD),^19^, ^20^ and some hypothesize that the notion of health care staff burnout and moral injury are related.^21^, ^22^, ^23^ Throughout the interviews, it became clear that participants regularly experienced or witnessed distress, either that of the patients and their relatives or their colleagues. PMIEs were not constrained to only the ‘witnessing distress’ category but weaved throughout the other categories in the data. An example of this was the distress witnessed by participants when discussing EoL care provisions. Evidence also suggests that recurrent exposure to PMIEs may impact care delivery and potentially dehumanize practice.^18^ This was also supported within this analysis, with participants describing the dehumanization of patients. These experiences are similar to those described in studies where staff are caring for patients with COVID‐19.^24^, ^25^ The present study focused on a population of health care staff caring for non‐COVID‐19‐related patients but subjected to the same restrictions as health care staff caring for those with COVID‐19. This suggests that ongoing support for staff following the COVID‐19 pandemic should not be solely focused on those practitioners caring for patients with COVID‐19 but should be available to all clinical staff.

In a paper from the British Medical Association^26^ discussing moral injury, they recognize the need to tackle the issue of moral injury within the National Health Service. They recommend taking a two‐pronged approach to supporting staff in order to lessen the potential for moral injury. They propose both organizational and individual recommendations, which include but are not limited to: adequate funding and resources; optimizing staffing levels; opening conversation about moral injury; and developing support networks for staff.^26^ These recommendations have been echoed in similar position statements from other organizations.^27^ It is evident from our research that had these strategies been in place, and frontline health care staff been allowed to take a more active role in decision‐making with regard to visiting, for example, during EoL care, it may have reduced their exposure to PMIEs and in turn reduced their potential for developing moral injury.

## 9 STUDY LIMITATIONS

The strengths of this study include the systematic implementation of grounded theory methodology and a robust audit trail of the data and analysis. However, there are also several limitations to this study. First, this is a single‐centre study in a tertiary care centre; therefore, the experiences described here may not be representative of all centres caring for non‐COVID‐19 patients as experiences may have differed. The sampling method adopted may have added potential for bias as participants self‐selected for inclusion in the study. Finally, rigorous approaches to analysis were undertaken; however, despite this, other interpretations of the data might be possible.

## 10 IMPLICATIONS AND RECOMMENDATIONS

Across health care systems, reducing the risk of moral injury to health care staff should be a priority because of its potential impact on staff as well as patients. Currently, most treatment strategies focus around one aspect of PMIEs, the management of PTSD,^20^ rather than a holistic approach across the spectrum of sequelae. Future work should investigate strategies that prevent moral injury as well as manging its manifestation. This is likely to require a tailored approach that recognizes individual coping. Moreover, environmental and organizational factors also require consideration, especially in relation to the delivery of EoL care and the implementation of policy and practice change.^28^

The data collected in this study provide a theory that health care staff caring for non‐COVID‐19 critical care patients during the period of visiting restrictions were exposed to PMIEs. Repeated exposure to PMIEs has the potential to lead to the development of moral injury and its adverse consequences. Priority should be placed on supporting all staff in the post‐pandemic era; this should include prioritizing research into the management of moral injury. Together with this, organizations should implement strategies to help reduce exposure to PMIEs in the future.

## FUNDING INFORMATION

LH was funded through a Royal College of Nursing Foundation (UK) bursary and JM was funded through The Healthcare Improvement Studies Institute Fellowship, University of Cambridge (PD‐2019‐02‐16).

## ETHICS STATEMENT

Ethical approval was obtained from the Yorkshire & The Humber – South Yorkshire Research Ethics Committee (20/YH/0236) prior to recruitment. All patients provided written, informed consent prior to participation.

## PARTICIPANT CONSENT STATEMENT

We participants received both written and verbal information about the study prior to giving written consent.

## Data Availability

A de‐identified dataset and the study protocol may be made available to researchers with a methodologically sound proposal, to achieve the aims described in the approved proposal. Data will be available upon request following article publication. Requests for data should be directed at joanne.mcpeake@glasgow.ac.uk to gain access.

## APPENDIX 1 SEMI‐STRUCTURED INTERVIEW GUIDE: VINCI

### 1.1

Health Care Staff.

1. You are a ______ here?
  - How long have you worked here?
  - And did you work here/anywhere else prior to COVID‐19 changing visiting?
2. Can you tell me more about your role in the ICU?
3. Could you describe how you in your professional role interact with visitors in the ICU?
4. How do you feel families are involved in the care of patients in the ICU prior to COVID‐19?
5. What impact has been on staff, with regard to visiting because of COVID‐19?
  - Is there any moments related to visitors and visiting that stood out to you?
6. Can you tell me how you feel how patients are affected by the lack of visitors during the COVID‐19 pandemic?
  - What were the negatives of this situation?
  - Did you feel there were positives to come from the lack of visitors during the pandemic?
  - Did you feel there were any positives for the patients?
  - Equally, can you describe any negatives for the patients?
  - How did you think relatives found this situation?
7. What was the impact on the communication with relatives?
  - How did you find this experience?
  - How did this affect your practice?
  - Did you feel this was significantly different to before COVID‐19?
8. How well did feel you knew your patients?
  - Do you feel visitors affect how you know your patient?
  - How did not having visitors affect how well you knew your patients?
9. Thinking about your recent experience, how has COVID‐19 changed your views about visiting?
10. Is there anything you think could have been done better with regard to visiting during COVID‐19?
11. Is there anything about your experience of working in critical care during COVID‐19 in relation to visitors and visiting that you think I should understand better?
12. Is there anything you would like to ask me?
13. Would you like to be informed of the results of this study?
